# Retinal and Choroidal Morphological Features Influencing Contrast Sensitivity in Retinitis Pigmentosa

**DOI:** 10.3390/medicina61091681

**Published:** 2025-09-17

**Authors:** Francisco de Asís Bartol-Puyal, Beatriz Cordón Ciordia, Elisa Viladés Palomar, Carlos Santana Plata, Silvia Méndez-Martínez, Luis Pablo Júlvez

**Affiliations:** 1 Ophthalmology Department, Miguel Servet University Hospital, 50009 Zaragoza, Spain; 2 Grupo Investigación Miguel Servet Oftalmología (GIMSO), Health Research Institute Aragón (IIS Aragón), 50009 Zaragoza, Spain; 3 Surgery Department, University of Zaragoza, 50009 Zaragoza, Spain; 4 Biotech Vision SLP (Spin-Off Company), University of Zaragoza, 50009 Zaragoza, Spain

**Keywords:** choroidal thickness, contrast sensitivity, optical coherence tomography, optical coherence tomography angiography, retinitis pigmentosa

## Abstract

*Background and Objectives*: To find morphological features on optical coherence tomography (OCT) and OCT-angiography (OCTA) influencing contrast sensitivity (CS) in patients with retinitis pigmentosa (RP). *Materials and Methods*: Cross-sectional study enrolling 18 eyes of 18 patients with RP. They were examined with CSV1000-E (VectorVision) under mesopic conditions (logarithmic scale), spectral-domain OCT (SD-OCT, Spectralis), swept-source OCT (SS-OCT, Triton), and OCTA (Triton). Automatic thickness measurements of every retinal layer were obtained in grids of 8 × 8 and 10 × 10 cubes. Foveal avascular zone and vascular densities (VD) were also analyzed. Statistical analysis included multiple lineal regression analyses, and a correlation analysis between age, axial length, and intraocular pressure, and retinal nerve fiber layer (RNFL) thickness. *Results*: Mean age was 47.34 ± 13.77 years. Mean CS with 3, 6, 12, and 18 cycles/degree (c/d) was 1.48 ± 0.37, 1.51 ± 0.39, 1.00 ± 0.42, and 0.44 ± 0.39, respectively. The most related variables to 3 c/d frequency were nasal RFNL thickness (R^2^ = 0.54) and central outer plexiform layer (OPL) (R^2^ = 0.33). In case of 6 c/d frequency, it was central VD in deep plexus (R^2^ = 0.66), and retinal pigment epithelium (RPE) (R^2^ = 0.22). As for 12 c/d frequency, it was central RNFL (R^2^ = 0.50), and central VD in deep plexus (R^2^ = 0.26). Regarding 18 c/d frequency, it was central RNFL (R^2^ = 0.70). *Conclusions*: Central and nasal RNFL thickness seem to be main predictors of CS in patients with RP, as well as VD in deep retinal plexus. Others with limited influence might be central and nasal OPL thickness, and central RPE thickness.

## 1. Introduction

Retinitis pigmentosa (RP) is an inherited retinal dystrophy (IRD) characterized by atrophy of photoreceptors in peripheral retina, and it may affect cones and fovea in late stages. As a consequence, retinal pigment epithelium (RPE) and choriocapillaris become atrophied, as well. More than one hundred genetic mutations have been described so far [1], and progression speed depends on them [2]. However, all of them show common signs and symptoms, such as reduced visual field in initial stages, and preserved best corrected visual acuity (BCVA) until last stages. Therefore, BCVA alone may not reflect real experience of patients because visual quality is usually affected before BCVA [3,4].

Thus, CS in patients with RP may show deficits even before BCVA drops. Previous studies have shown that both are correlated. RP shows larger CS deficits with BCVA reduction, in comparison with other pathologies [5]. It has also been proven that CS decreases more in mesopic than in photopic conditions [6].

Although photoreceptor and RPE atrophy have been documented deeply, and progressive decrease in visual quality has also been reported, correlation between both has been studied to a lesser extent [7,8]. Thus, the purpose of this study is to find morphological features with an influence on CS in patients with RP, as part of the study of the structure-function relationship in these patients.

## 2. Methods

Patients with RP were enrolled in this cross-sectional study. Inclusion criteria were age older than 18 years, RP with genetics confirmation, BCVA ≥ 0.2 in decimal scale (20/100 in Snellen scale, 0.70 logMAR), and an axial length (AL) between 22 and 26 mm. Exclusion criteria included any other ophthalmological disorder such as ocular hypertension or glaucoma, or diabetic retinopathy among others, previous ophthalmological surgery, any other systemic condition that may alter ophthalmological outcomes, cataracts, pregnancy and puerperium, and systemic treatment with corticoids or biologic therapies, and any acute infection. The only exception was cataracts surgery if it had been performed three or more months earlier. This study was approved by the local ethics committee (PI23/467) and adhered to the tenets of the Declaration of Helsinki. All patients were informed about the study and all of them signed the informed consent.

A deep ophthalmological examination was performed by an experienced retina subspecialist including refractometry, BCVA with ETDRS charts at a 6 m distance under photopic lighting conditions (10 cd/m^2^), slit-lamp examination, Goldmann tonometry, and fundus examination after pharmacological mydriasis with topical tropicamide. In case the patient was included in the study, further tests were performed between one and seven days afterwards. All tests were performed in both eyes, but only the eye with worst BCVA was selected for statistical analysis.

CS was examined with CSV-1000E test (VectorVision, Greenville, OH, USA) at a 4 m distance under mesopic conditions (3 cd/m^2^) with optical correction monocularly. This test consists of four rows of circles with 3, 6, 12, and 18 cycles/degree (c/d) frequencies. These are presented in pairs of circles, one with contrast and the other being plane. The contrast in every frequency reduces progressively, and the patient has to say which circle presents contrast. Outcomes were taken on logarithmic scale, as provided by the designer.

Afterwards, their pupils were dilated with topical tropicamide. All the following tests were performed under scotopic conditions (0 cd/m^2^). First, they were examined with IOLmaster500 (Carl Zeiss, Jena, Germany) in order to obtain their AL. Then, retinal was imaged with spectral-domain optical coherence tomography (SD-OCT) Spectralis (Heidelberg Engineering, Heidelberg, Germany). A fovea-centered macular analysis of 6 × 6 mm and 17 scans was performed. Later, they were examined with swept-source OCT (SS-OCT) deep range imaging (DRI) Triton (software version 1.1.7, Topcon Corporation, Tokyo, Japan). A fovea-centered macular of 7 × 7 mm was performed, and then retinal vasculature was analyzed with OCT-angiography (OCTA) using the same device. The size of the OCTA acquisition was 6 × 6 mm.

Spectralis OCT makes automatic segmentation of retinal layers and gives their thickness in a grid of 8 × 8 cubes within the 6 × 6 mm acquisition. Similarly, Triton OCT makes automatic segmentation of some retinal layers, as well as for the choroid, and gives their thickness in a grid of 10 × 10 cubes. Therefore, using Spectralis OT we obtained thickness of the whole retina, retinal nerve fiber layer (RNFL), ganglion cell layer (GCL), inner plexiform layer (IPL), inner nuclear layer (INL), outer plexiform layer (OPL), outer nuclear layer (ONL), and RPE. We obtained choroidal thickness (CT) using Triton OCT. The OCTA makes automatic measurements of foveal avascular zone (FAZ) and vascular densities (VD) in superficial and deep retinal plexus.

Other data were recorded for posterior statistical analysis. These included age and type of genetic mutation. The latter was coded into a qualitative variable, with three different categories: mutations with autosomal dominant (AD) inheritance, mutations with autosomal recessive (AR) inheritance, and other or mixed types of mutations.

Statistical analysis was performed using the Statistical Package for the Social Sciences (SPSS) software for Windows (software version 20, IBM Corporation, Somers, NY, USA). Mean and standard deviations were calculated for quantitative variables, as well as minimal and maximal values. Multiple regression analyses were performed, establishing 3, 6, 12, and 18 c/d frequencies of the CS test as the dependent variables. Independent variables were age, AL, IOP, type of genetic mutation, and thickness of every grid sector of every retinal layer and of the choroid. Bivariate correlation analysis (Spearman test) was performed between age, AL, and IOP on one side, and RNFL thickness in every location on the other side. Statistical significance was established at *p* < 0.05.

## 3. Results

A total of 18 eyes of 18 patients with RP were included in the study. Mean age was 47.34 ± 13.77 years (range 26.68–75.05), mean AL was 23.81 ± 1.03 mm (range 22.02–25.77), mean BCVA was 0.14 ± 0.17 in the logMAR scale (range −0.10–0.50), mean IOP was 16.41 ± 2.65 mmHg (range 11–21). A total of 8 patients were male, and 10 were female. There were 8 right eyes, and 10 left eyes. In all, 4 patients had AD mutations, 8 had AR mutations, and 6 had mixed types of mutations. Table 1 displays demographic data, along with genetic mutations.

**Table 1:** Demographic data.

Patient	Gender	Age, Years	Eye	BCVA, logMAR	Genetic Mutations
1	Female	57	Right	−0.10	RP1—c.2289_2299del (p.Asn763LysfsTer14)—AD
2	Female	44	Left	0.02	IFT140—c.932A > G (p.Tyr311Cys)—AR IMPG2—c.2906A > G (p.Asn969Ser)—AR
3	Male	42	Right	0.00	PRPF3—c.1481C > T (p.Thr494Met)—AD
4	Male	31	Right	0.10	USH2A—c.2431_2432del (p.Lys811Aspfs*11)—AR USH2A—c.2299del (p.Glu767Serfs*21)—AR
5	Male	67	Left	0.12	USH2A—c.2299del (p.Glu767Serfs*21)—AR USH2A—c.2276G > T (p.?)—AR
6	Female	62	Right	0.18	CNGA1—c.859C > T (p.Arg287*)—AR CNGA1—c.1166C > T (p.Ser259Phe)—AR ABCA4—c.5461-10T > C (p.?)—AR
7	Male	28	Left	0.22	RP1L1—c.1435G > A (p.Gly479Arg)—AD
8	Male	53	Right	0.12	CNGB1—c.2957A > T (p.Asn986Ile)—AR CNGB1—c.2957A > T (p.Asn986Ile)—AR
9	Female	27	Left	0.00	ABCA4—c.4253 + 43G > A (p.?)—AR USH2A—c.7906A > G (p.Thr2636Ala)—AR
10	Female	53	Left	0.30	MYO7A—c.395C > T (p.Pro132Leu)—AR MYO7A—c.395C > T (p.Pro132Leu)—AR ABCA4—c.5882G > A (p.Gly1961Glu)—AR
11	Female	32	Right	0.14	RHO—c.568G > T (p.Asp190Tyr)—AD
12	Male	40	Left	0.46	MERTK—c.2201A > T (p.Asp734Val)—AR RGR—c.394G > A (p.Val132Met)—AD
13	Female	52	Left	0.10	CNGB1—c.2957A > T (p.Asn986Ile)—AR USH2A—g.216051172G > A (p.Pro2870Leu)—AR
14	Male	33	Right	0.50	PDE6B—c.905G > A (p.Gly302Asp)—AD
15	Female	53	Left	0.03	EYS—c.6079-2A > G (p.?)—AR EYS—c.6079-2A > G (p.?)—AR
16	Female	75	Right	0.28	RP1—c.2207del (p.Thr736Metfs*2)—AD
17	Male	49	Left	0.02	USH2A—c.2167 + 5G > A (p.?)—AR USH2A—c.12569T > A (p.Val4190Glu)—AR
18	Female	51	Right	0.00	USH2A—c.2276G > T (p.Cys759Phe)—AR USH2A—c.12280A > G (p.Asn4094Asp)—AR

BCVA = best corrected visual acuity, AD = autosomal dominant, AR = autosomal recessive. Mean CS in logarithmic scale with 3 c/d was 1.48 ± 0.37 (range 0.70–1.93), with 6 c/d it was 1.51 ± 0.39 (range 0.91–2.14), with 12 c/d it was 1.00 ± 0.42 (range 0.31–1.84), and with 18 c/d it was 0.44 ± 0.39 (range −0.13–1.10). Mean SD-OCT Spectralis quality was 28.44 ± 3.78 (range 21–34). Mean SS-OCT Triton quality was 61.72 ± 7.82 (range 43.53–70.68). Retinal and CT are displayed in Table 2 and Table 3.

**Table 2:** Mean thickness in every retinal layer with SD-OCT Spectralis in a grid of 8 × 8 cubes.

Retina, μm	1	2	3	4	5	6	7	8
1	234.28 ± 34.98	234.78 ± 40.72	245.72 ± 46.82	249.78 ± 38.81	261.83 ± 38.70	284.00 ± 46.01	304.61 ± 52.33	304.65 ± 58.05
2	232.06 ± 43.32	234.17 ± 44.93	241.44 ± 39.45	253.78 ± 42.80	262.67 ± 41.99	268.33 ± 45.37	284.56 ± 44.93	315.72 ± 48.13
3	229.22 ± 39.83	234.11 ± 38.15	262.00 ± 44.63	292.33 ± 46.29	301.39 ± 47.01	292.22 ± 51.14	278.28 ± 52.70	291.56 ± 47.60
4	225.44 ± 39.36	246.17 ± 40.77	288.11 ± 46.08	293.06 ± 44.55	283.11 ± 47.01	316.33 ± 47.26	292.00 ± 50.20	267.06 ± 44.83
5	217.94 ± 36.18	241.11 ± 40.28	281.61 ± 47.46	286.00 ± 46.06	286.28 ± 43.16	322.78 ± 48.61	293.56 ± 56.75	263.67 ± 50.75
6	210.78 ± 36.88	228.39 ± 40.27	262.72 ± 49.12	294.33 ± 53.64	306.94 ± 53.35	300.06 ± 57.14	277.33 ± 58.59	275.67 ± 54.98
7	207.78 ± 38.29	214.56 ± 39.68	235.61 ± 47.02	259.22 ± 55.29	268.22 ± 55.55	264.33 ± 51.82	264.44 ± 47.85	286.94 ± 51.04
8	215.72 ± 34.86	220.61 ± 36.22	227.89 ± 44.11	240.50 ± 46.13	250.17 ± 47.97	254.33 ± 45.21	270.67 ± 41.88	290.12 ± 41.74
RNFL, μm	1	2	3	4	5	6	7	8
1	22.72 ± 10.13	24.56 ± 13.42	29.50 ± 17.76	32.44 ± 20.35	34.83 ± 24.33	48.00 ± 30.25	64.89 ± 38.94	64.94 ± 42.59
2	21.22 ± 10.35	23.06 ± 11.76	25.78 ± 14.05	27.00 ± 15.99	29.56 ± 17.21	35.89 ± 22.34	56.83 ± 34.35	73.06 ± 44.31
3	18.72 ± 9.05	18.78 ± 9.14	20.94 ± 13.13	23.22 ± 14.86	25.17 ± 15.76	30 ± 18.31	39.22 ± 21.00	65.33 ± 31.25
4	14.61 ± 4.91	14.72 ± 5.04	16.39 ± 5.25	15.56 ± 4.55	12.00 ± 5.31	18.72 ± 8.70	25.94 ± 15.34	40.28 ± 21.84
5	13.78 ± 4.22	14.11 ± 3.92	14.00 ± 4.69	13.94 ± 4.17	12.44 ± 5.18	21.89 ± 10.10	29.17 ± 17.80	38.72 ± 25.20
6	14.67 ± 4.84	14.56 ± 5.32	19.06 ± 10.71	24.78 ± 16.78	28.22 ± 19.32	32.00 ± 19.96	39.00 ± 25.87	54.67 ± 34.37
7	17.22 ± 7.73	18.78 ± 10.24	21.72 ± 13.86	27.06 ± 18.15	31.83 ± 22.72	36.11 ± 25.50	46.28 ± 30.95	62.11 ± 41.41
8	19.28 ± 7.67	21.17 ± 9.92	23.06 ± 14.09	28.06 ± 17.97	31.94 ± 22.30	37.17 ± 26.15	50.50 ± 32.25	60.76 ± 40.74
GCL, μm	1	2	3	4	5	6	7	8
1	18.00 ± 7.45	19.61 ± 8.75	20.72 ± 7.78	22.89 ± 9.72	27.33 ± 11.70	29.67 ± 12.96	29.61 ± 11.77	28.76 ± 10.30
2	16.56 ± 6.99	18.17 ± 7.32	21.11 ± 8.34	24.06 ± 10.03	25.78 ± 10.59	26.17 ± 10.41	25.94 ± 9.36	30.61 ± 10.06
3	15.94 ± 9.02	18.94 ± 12.61	23.11 ± 15.68	26.89 ± 19.22	27.94 ± 19.53	27.67 ± 16.14	26.39 ± 9.59	27.39 ± 4.94
4	16.5 ± 11.49	22.61 ± 16.62	32.22 ± 22.86	31.67 ± 19.35	22.83 ± 14.35	31.72 ± 22.51	27.83 ± 17.11	24.50 ± 7.93
5	19.17 ± 10.96	26.28 ± 15.03	32.22 ± 22.05	29.06 ± 17.43	23.78 ± 14.24	36.44 ± 22.84	31.00 ± 16.79	28.39 ± 9.60
6	17.06 ± 10.35	22.50 ± 15.25	26.06 ± 19.36	29.83 ± 21.57	30.94 ± 20.60	30.56 ± 17.02	27.67 ± 11.28	27.28 ± 7.20
7	14.61 ± 7.16	16.44 ± 8.25	20.5 ± 11.25	25.94 ± 16.38	27.56 ± 14.75	26.89 ± 10.38	26.22 ± 7.38	28.22 ± 8.02
8	15.72 ± 6.27	17.33 ± 6.70	19.11 ± 8.48	21.33 ± 8.57	23.33 ± 9.22	24.39 ± 9.21	26.39 ± 10.97	30.12 ± 12.33
IPL, μm	1	2	3	4	5	6	7	8
1	25.28 ± 8.90	26.78 ± 9.59	28.44 ± 12.82	30.22 ± 12.66	35.39 ± 15.08	38.44 ± 18.67	38.61 ± 14.76	36.65 ± 14.54
2	22.78 ± 7.36	24.67 ± 7.53	26.11 ± 7.50	29.17 ± 8.71	32.06 ± 8.97	35.11 ± 12.85	35.83 ± 17.41	41.06 ± 17.50
3	21.50 ± 6.09	22.89 ± 7.25	25.83 ± 9.15	28.06 ± 10.64	29.56 ± 10.97	31.67 ± 8.74	33.94 ± 3.98	35.06 ± 8.10
4	19.44 ± 7.17	22.06 ± 10.38	28.39 ± 15.95	29.78 ± 12.37	24.5 ± 10.67	31.28 ± 15.06	30.06 ± 9.26	32.94 ± 6.95
5	19.17 ± 7.63	23.83 ± 11.61	29.22 ± 15.46	27.72 ± 12.56	25.22 ± 10.08	34.44 ± 16.90	32.39 ± 9.67	36.78 ± 10.6
6	19.06 ± 5.95	20.67 ± 7.83	25.00 ± 10.82	28.00 ± 12.68	29.83 ± 11.14	31.83 ± 8.02	34.50 ± 4.88	34.39 ± 9.08
7	18.78 ± 4.92	20.06 ± 5.41	23.94 ± 7.82	28.94 ± 11.80	31.89 ± 9.46	33.00 ± 7.87	33.22 ± 8.59	35.67 ± 12.85
8	20.72 ± 5.55	23.28 ± 7.47	24.83 ± 8.87	28.28 ± 10.07	30.00 ± 10.57	31.61 ± 11.92	33.78 ± 13.55	38.00 ± 17.06
INL, μm	1	2	3	4	5	6	7	8
1	19.28 ± 6.01	19.56 ± 5.44	22.33 ± 8.52	23.06 ± 8.84	24.39 ± 9.43	24.28 ± 6.85	24.44 ± 6.37	24.82 ± 7.34
2	21.39 ± 6.94	23.50 ± 7.11	26.72 ± 6.94	29.44 ± 6.26	31.11 ± 6.97	28.61 ± 7.05	24.83 ± 7.70	24.78 ± 8.48
3	22.06 ± 5.91	28.61 ± 6.24	38.44 ± 8.85	47.83 ± 9.13	48.61 ± 9.68	42.83 ± 10.10	31.89 ± 10.45	24.89 ± 7.78
4	23.56 ± 7.79	32.83 ± 9.03	45.89 ± 12.03	42.22 ± 10.85	35.22 ± 9.82	51.28 ± 9.44	45.22 ± 9.00	28.50 ± 7.05
5	20.28 ± 6.91	29.06 ± 8.99	41.56 ± 9.37	38.61 ± 7.75	38.11 ± 10.64	52.67 ± 8.12	44.44 ± 8.37	26.72 ± 8.98
6	20.56 ± 6.43	27.61 ± 7.27	39.22 ± 8.87	49.78 ± 9.67	48.67 ± 7.57	44.72 ± 9.37	32.72 ± 10.58	24.89 ± 8.46
7	19.06 ± 6.24	22.89 ± 6.58	30.78 ± 8.38	33.89 ± 8.28	33.44 ± 7.01	29.78 ± 6.75	24.78 ± 5.97	22.94 ± 5.96
8	19.33 ± 5.97	20.83 ± 5.90	22.28 ± 7.47	23.56 ± 9.00	24.61 ± 9.94	24.28 ± 8.56	22.72 ± 6.32	22.24 ± 7.37
OPL, μm	1	2	3	4	5	6	7	8
1	20.22 ± 4.40	22.44 ± 5.09	23.28 ± 3.58	24.00 ± 3.48	24.56 ± 4.62	24.22 ± 5.00	23.11 ± 4.86	21.47 ± 3.68
2	22.39 ± 4.46	24.94 ± 3.93	28.67 ± 3.56	31.39 ± 5.24	31.22 ± 3.75	30.56 ± 4.55	27.00 ± 5.06	23.94 ± 4.50
3	24.33 ± 3.83	28.94 ± 2.86	34.94 ± 5.09	38.78 ± 6.31	40.22 ± 6.43	39.22 ± 6.84	34.72 ± 6.79	27.44 ± 5.94
4	27.72 ± 4.16	34.00 ± 5.83	36.67 ± 5.04	33.61 ± 8.66	36.50 ± 12.32	44.44 ± 11.90	39.56 ± 6.12	30.78 ± 4.97
5	26.17 ± 5.72	31.78 ± 5.56	36.33 ± 5.99	34.17 ± 6.36	35.22 ± 8.76	41.89 ± 8.30	36.78 ± 5.05	28.39 ± 2.68
6	25.61 ± 2.87	31.17 ± 4.15	35.11 ± 6.13	35.50 ± 4.42	38.39 ± 4.83	36.94 ± 4.50	32.67 ± 4.06	27.11 ± 3.01
7	23.22 ± 3.83	26.56 ± 4.19	28.72 ± 3.69	31.00 ± 4.52	31.28 ± 4.35	30.17 ± 4.31	26.17 ± 1.82	23.83 ± 3.81
8	20.61 ± 3.68	22.72 ± 3.71	23.78 ± 5.55	24.50 ± 4.49	24.89 ± 4.06	23.17 ± 3.99	22.50 ± 3.78	20.94 ± 4.78
ONL, μm	1	2	3	4	5	6	7	8
1	41.33 ± 10.52	37.83 ± 11.62	37.39 ± 14.87	36.39 ± 11.79	34.00 ± 10.57	36.39 ± 13.8	38.50 ± 14.57	39.59 ± 19.40
2	40.22 ± 14.86	37.61 ± 14.83	34.61 ± 12.02	34.94 ± 11.42	35.44 ± 10.68	33.78 ± 12.02	32.61 ± 11.99	34.67 ± 11.86
3	41.78 ± 14.35	38.44 ± 12.49	41.17 ± 10.30	46.00 ± 9.43	47.00 ± 10.55	40.50 ± 10.41	33.89 ± 12.91	30.17 ± 11.78
4	40.94 ± 15.16	41.50 ± 11.82	48.33 ± 13.25	58.44 ± 16.38	68.94 ± 16.20	56.33 ± 16.95	43.67 ± 15.53	30.50 ± 11.14
5	39.83 ± 14.05	39.11 ± 9.52	48.33 ± 12.11	60.44 ± 14.98	68.78 ± 14.35	54.00 ± 12.13	40.83 ± 10.31	27.00 ± 10.11
6	35.78 ± 12.28	36.17 ± 10.47	40.67 ± 11.07	45.50 ± 12.07	48.00 ± 12.54	45.00 ± 11.67	33.33 ± 12.38	28.56 ± 13.33
7	34.44 ± 12.33	33.28 ± 13.06	34.33 ± 13.47	35.33 ± 13.22	35.67 ± 12.90	32.44 ± 13.51	30.56 ± 13.36	31.28 ± 12.39
8	36.50 ± 11.78	35.06 ± 11.87	35.11 ± 12.63	35.28 ± 12.80	34.72 ± 12.78	32.33 ± 12.60	32.67 ± 12.07	32.41 ± 12.18
RPE, μm	1	2	3	4	5	6	7	8
1	20.11 ± 10.53	17.33 ± 7.51	17.44 ± 7.92	15.39 ± 6.05	16.78 ± 7.31	18.67 ± 13.99	21.22 ± 19.65	24.18 ± 25.06
2	21.44 ± 13.98	17.06 ± 8.57	13.72 ± 4.87	13.39 ± 3.40	12.83 ± 1.54	13.33 ± 3.24	16.44 ± 11.72	21.44 ± 20.65
3	19.17 ± 10.74	12.83 ± 4.19	11.78 ± 1.26	12.61 ± 0.92	12.89 ± 0.96	12.67 ± 1.14	12.33 ± 0.97	15.33 ± 6.53
4	17.17 ± 10.09	12.5 ± 2.87	12.61 ± 1.33	13.83 ± 1.72	15.33 ± 2.30	13.72 ± 1.60	12.72 ± 1.49	14.33 ± 9.28
5	14.39 ± 6.51	11.61 ± 1.46	12.83 ± 1.34	14.00 ± 2.03	15.06 ± 2.31	13.89 ± 1.41	12.28 ± 1.23	12.17 ± 2.12
6	14.39 ± 9.27	11.39 ± 1.69	12.28 ± 1.49	13.83 ± 3.35	14.83 ± 4.30	13.33 ± 2.09	12.61 ± 1.33	14.00 ± 4.90
7	15.67 ± 8.39	12.72 ± 3.97	11.56 ± 1.62	12.33 ± 1.81	12.28 ± 1.07	12.11 ± 1.75	13.67 ± 4.16	19.44 ± 17.49
8	18.39 ± 12.18	15.44 ± 7.30	14.11 ± 5.13	14.17 ± 5.84	16.22 ± 8.52	17.06 ± 12.78	18.39 ± 17.33	22.35 ± 24.33
**The color-coded grid leyend is the following:**								
**Color**	**Retina, μm**	**RNFL, μm**	**GCL, μm**	**IPL, μm**	**INL, μm**	**OPL, μm**	**ONL, μm**	**RPE, μm**
Red	<225	<20	<20	<20	<25	<20	<35	<5
Orange	225–249	20–29	20–24	20–24	25–34	20–24	35–39	5–9
Yellow	250–274	30–39	25–29	25–29	35–44	25–29	40–44	10–14
Green	275–299	40–49	30–34	30–34	45–54	30–34	45–49	15–19
Blue	≥300	≥50	≥35	≥35	≥55	≥35	≥50	≥20

RNFL = Retinal Nerve Fiber Layer, GCL = Ganglion Cell Layer, IPL = Inner Plexiform Layer, INL = Inner Nuclear Layer, OPL = Outer Plexiform Layer, ONL = Outer Nuclear Layer, RPE = Retinal Pigment Epithelium.

**Table 3:** Mean choroidal thickness with SS-OCT Triton in a grid of 10 × 10 cubes.

CT, μm	1	2	3	4	5	6	7	8	9	10
1	178.75 ± 82.46	191.24 ± 82.76	204.19 ± 80.75	217.21 ± 79.08	224.32 ± 82.89	222.76 ± 79.08	214.72 ± 75.85	205.11 ± 82.42	195.45 ± 85.79	189.09 ± 83.28
2	169.86 ± 75.75	182.58 ± 81.93	197.64 ± 81.99	211.63 ± 86.60	220.62 ± 87.90	222.96 ± 83.59	219.29 ± 78.39	207.96 ± 75.63	198.38 ± 69.92	198.07 ± 61.76
3	166.45 ± 76.77	177.28 ± 80.35	192.31 ± 84.01	210.37 ± 84.84	218.72 ± 85.62	224.53 ± 92.27	221.65 ± 97.35	203.15 ± 95.33	188.87 ± 91.14	181.32 ± 81.12
4	167.81 ± 74.3	183.76 ± 74.25	203.00 ± 80.71	218.46 ± 88.69	227.01 ± 91.22	225.97 ± 94.65	213.10 ± 96.00	192.77 ± 97.11	200.01 ± 111.75	189.66 ± 108.78
5	183.44 ± 95.67	198.95 ± 89.62	214.98 ± 86.10	227.26 ± 86.42	232.25 ± 90.19	231.29 ± 92.50	217.44 ± 87.32	199.50 ± 86.40	178.57 ± 87.98	162.29 ± 81.43
6	175.63 ± 89.83	192.37 ± 80.91	208.35 ± 76.37	220.20 ± 83.31	223.59 ± 89.86	225.85 ± 86.94	218.88 ± 81.61	202.82 ± 85.31	183.10 ± 89.14	167.76 ± 85.56
7	172.05 ± 97.36	181.77 ± 87.36	198.27 ± 79.52	218.60 ± 81.51	232.95 ± 87.95	236.25 ± 89.31	231.65 ± 81.55	219.85 ± 77.17	199.32 ± 76.51	186.07 ± 76.41
8	167.68 ± 97.86	175.49 ± 94.69	189.35 ± 89.40	212.29 ± 86.63	229.89 ± 88.58	236.11 ± 85.90	226.45 ± 79.83	209.65 ± 78.86	190.16 ± 80.00	172.34 ± 76.23
9	167.28 ± 88.91	172.83 ± 82.23	185.34 ± 75.17	204.15 ± 72.48	220.94 ± 79.20	227.30 ± 79.94	222.20 ± 78.16	207.59 ± 75.17	186.74 ± 77.44	171.90 ± 79.27
10	157.36 ± 77.52	168.36 ± 72.50	180.14 ± 71.08	195.95 ± 77.01	209.45 ± 81.35	212.29 ± 79.05	201.39 ± 74.90	182.94 ± 72.83	164.97 ± 71.09	155.01 ± 73.94

CT = Choroidal Thickness. Red highlights CT lower than 174 μm, orange highlights CT between 175 and 199 μm, yellow highlights CT between 200 and 224 μm, and green highlights CT higher than 225 μm.

**Figure 1:**
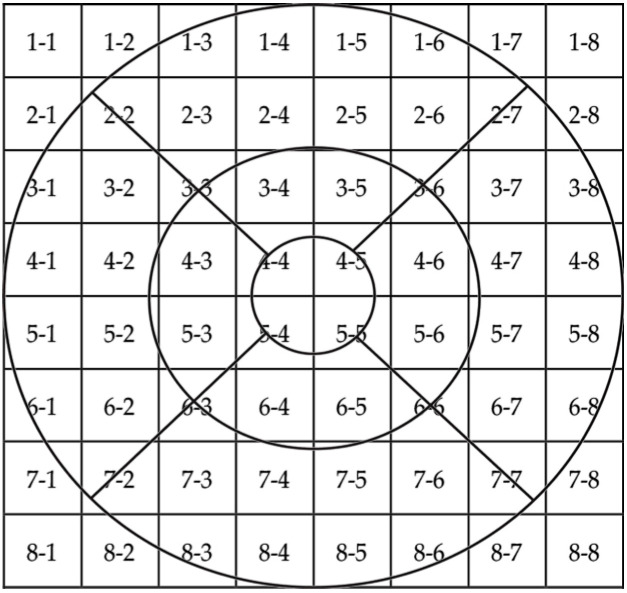
Superposition of the 8 × 8 grid and the ETDRS.

Regression analyses were statistically significant for all spatial frequencies. When reporting retinal or choroidal locations, the first number corresponds to the row, and the second number corresponds to the column within the grid in a right eye model. Figure 1 shows the 8 × 8 grid together with ETDRS grid for a better understanding. Size grid in SD-OCT is 8 × 8, and in SS-OCT is 10 × 10. 3 c/d frequency outcomes were predicted by the following variables: RNFL in 3-8 location (R^2^ = 0.40, β = 0.010, CI 95% [0.005, 0.014]) and OPL in 3-2 location (R^2^ = 0.33, β = 0.078, CI 95% [0.034, 0.122]). The following variables predicted 6 c/d frequency outcomes: central VD in deep retinal plexus (R^2^ = 0.60, β = −0.034, CI 95% [−0.046, −0.021]) and RPE in 5-4 location (R^2^ = 0.23, β = 0.64, CI 95% [0.037, 0.139]). The following variables predicted 12 c/d frequency outcomes: central VD in deep retinal plexus (R^2^ = 0.48, β = −0.02, CI [−0.031, −0.011]), RNFL in 4-4 location (R^2^ = 0.25, β = 0.04, CI 95% [0.020, 0.064]), OPL in 6-4 location (R^2^ = 0.15, β = 0.05, CI 95% [0.028, 0,069]), and RPE in 5-2 location (R^2^ = 0.06, β = 0.10, CI 95% [0.022, 0.182]). RNFL in 4-4 location (R^2^ = 0.69, β = 0.073, CI 95% [0.045, 0.101]) predicted 18 c/d frequency outcomes. Table 4 includes a brief summary of these outcomes. Age, AL, IOP, IPL, and INL thickness showed no statistical influence on CS outcomes (*p* > 0.05).

## 4. Discussion

The structural assessment of retina in our study was based on automatic measurements performed by the OCT software (v7.0.4). Although manual measurements can be considered reliable [9], automatic measurements have the highest repeatability and reproducibility [10]. SD-OCT and SS-OCT are both adequate for retinal imaging, but they use different wavelengths, so their outcomes are not interchangeable [11]. We also assessed retina and choroid in the whole macula, and not only within the limits of the ETDRS grid. This can be adequate for simple analyses, but a deep study should include a detailed mapping of the entire macula because some information could be omitted otherwise [12,13]. Previous studies compared structure and function in patients with RP, but these usually considered visual field or electroretinogram as visual quality. Nevertheless, correlation using tests such as contrast sensitivity or color perception has been studied to a lesser extent [7,8]. As far as we know, this is the first study trying to establish a relationship between CS and findings on OCTA in patients with RP.

The structure–function relationship in RP has been studied to a limited extent so far. In our study, we found that morphological changes in central retina account for most of these changes in CS. Some of the main responsible factors may be RNFL thickness and central VD in deep retinal plexus. OPL thickness might have some influence to some extent.

Although similar, some differences should be highlighted between different spatial frequencies. In general lines, all spatial frequencies could be predicted after central RNFL thickness and VD in deep retinal plexus. However, 3 c/d is rather related to central-nasal RNFL thickness, and 6 c/d is not related to this retinal layer. Thickness of OPL or RPE may have some influence on CS, but to a lesser extent, and future studies with bigger samples should confirm whether OPL and RPE have any influence on CS.

RP is an IRD featured by progressive peripheral photoreceptor loss, with secondary atrophy or RPE, choriocapillaris and other retinal layers. Therefore, it should be expected to find CS being dependent on RPE or ONL thickness. However, this only applied for CS with 6 c/d. In other spatial frequencies, RNFL was the major influencing factor, as in other disorders such as glaucoma [14]. Our explanation for this is that ONL and RPE are the first to be affected and therefore some degree of CS disfunction may appear initially. Major CS affection may occur as a consequence of secondary retinal atrophy.

Yioti et al. [8] performed a similar study, in which they correlated CS with retinal OCT findings. Main differences are that they included both eyes of most of the enrolled patients, that they did not perform OCTA, that they only analyzed the central subfield retinal thickness, cube average thickness, and the IS/OS junction length on OCT, and that they only performed correlation analyses, instead of regression analyses. They found the highest correlation between IS/OS junction length and CS. To some extent, these outcomes are similar to ours because we found OPL and RPE thickness to be predictors of CS outcomes.

Xiao et al. [15] correlated CS and ONL thickness in knockout mice. Main limitations are that they only measured ONL, but no other retinal layers as we did. Thus, outcomes are not directly comparable between both studies.

In contrast to our outcomes, Burstedt et al. [16] found no correlation between OCT and visual function in a five-year prospective study with patients with RP caused by RLBP1 mutation. In our sample size, none of our enrolled patients had this genetic mutation. In addition, they only analyzed the central retinal thickness using ETDRS grid, and not the entire macula as we did.

Jacobson et al. [2] compared retinal structure and visual function in patients RP. They found that patients with recessive mutations preserved retinal regions with normal structure and function. On the contrary, we could not find the type of mutation as a predictor of CS outcomes. The limited sample size of our study would be likely responsible for this.

There are some other previous studies evaluating CS in patients with RP, but usually with other types of test, and they did not analyze retinal thickness on OCT [4,6]. Those tests used in previous studies only explore one spatial frequency with decreasing contrast. In contrast, CSV-1000E explores four different spatial frequencies. In addition, they did not try to correlate CS outcomes with any retinal layer thickness.

Although little research has been conducted so far regarding structure–function relationship in inherited retinal dystrophies, it has been more deeply studied in other pathologies such as age-related macular degeneration (AMD) or glaucoma. In case of AMD, CS is reduced in intermediate AMD, but not in early AMD [17]. In case of neovascular AMD, CS can improve with antiVEGF treatment, and it is correlated with morphological features on OCT [18]. Bennett et al. studied the structure–function relationship in intermediate-late AMD, and found that a higher central RPE volume and a greater number of intraretinal hyperreflective foci (HRF) were associated with reduced CS at 6 to 12 c/d. Additionally, ONL thinning in the inner ring of the ETDRS grid and a greater number of HRF were associated with reduced CS at 1 and 3 c/d, too [19]. Retinal affections in AMD starts in choriocapillaris, RPE and photoreceptors layers, which is somewhat similar to RP. As a consequence, we found RPE and OPL to be minor predictors of CS in RP. Nevertheless, both studies are not directly comparable because they did not use OCTA, and their sample of patients was much higher than ours. Fatehi et al. conducted a similar study in patients with glaucoma, and found that 6 c/d spatial frequency was more associated with retinal thickness changes than others [20]. In contrast to our study, we found that 6 c/d spatial frequency was highly associated with VD in deep capillary plexus, but they did not use OCTA.

In regard to glaucoma, structure-function has been deeply analyzed so far. It is well known that when glaucoma progresses, both may deteriorate, but with differences in quantity of decrease and the time it occurs. Pang et al. evaluated the association between CS and structural damage in primary open glaucoma (POAG). They found that CS was associated with peripapillary RNFL thickness, radial peripapillary capillary density, macular GCL thickness and superficial macular vessel density [21]. Their study is somehow comparable to ours because they used both OCT and OCTA, although CS test was different. Our findings are similar to theirs, that is, RNFL thickness plays an important role in CS, as well as retinal vascular density. Nonetheless, their study was focused on peripapillary retinal thicknesses, and they did not evaluate external retinal layers as we did.

The strengths of our study are that we only included one eye per patient, and that it is the first study trying to establish relationships between visual quality and OCTA in patients with RP. Furthermore, very few previous studies have analyzed thicknesses of all retinal layers, and none of them have analyzed retinal thickness outside the limits of the ETDRS grid. Main limitation of our study is the limited sample size and that patients were affected with different genetic mutations. Therefore, our outcomes should be considered as a pilot study, and further research with a higher number of participants should be performed.

## 5. Conclusions

In conclusion, our outcomes suggest that central and nasal RNFL thickness may be the main predictors of CS in patients with RP, as well as VD in deep retinal plexus. Other predictors with limited influence might be central and nasal OPL thickness, and central RPE thickness. GCL, ONL, and CT have a minimal or no influence on CS.

## Figures and Tables

**Table 4:** Regression analyses outcomes.

Spatial Frequency	3 c/d	6 c/d	12 c/d	18 c/d
R^2^ ≥ 0.60		VD deep center		RNFL 4-4
R^2^ ≥ 0.50				
R^2^ ≥ 0.40	RNFL 3-8		VD deep center	
R^2^ ≥ 0.30	OPL 3-2			
R^2^ ≥ 0.20		RPE 5-4	RNFL 4-4	
R^2^ ≥ 0.10			OPL 6-4	
R^2^ < 0.10			RPE 5-2	

c/d = cycles/degree, RNFL = Retinal Nerve Fiber Layer, OPL = Outer Plexiform Layer, VD = vascular density. Numbers represent the location within the grid in a right eye model. The first number corresponds to the row, and the second number corresponds to the column. The correlation analysis between age, AL, and IOP on one side, and RNFL thickness in every location on the other side, showed no significant correlations (*p* > 0.05).

## Data Availability

The original contributions presented in the study are included in the article, further inquiries can be directed to the corresponding authors.
